# The Rho GTPase RhoB regulates cadherin expression and epithelial cell-cell interaction

**DOI:** 10.1186/s12964-015-0085-y

**Published:** 2015-01-29

**Authors:** Francisco M Vega, Mairian Thomas, Nicolas Reymond, Anne J Ridley

**Affiliations:** Randall Division of Cell and Molecular Biophysics, King’s College London, New Hunt’s House, Guy’s Campus, London, SE1 1UL UK; Current address: Instituto de Biomedicina de Sevilla, Hospital Universitario Virgen del Rocío/CSIC/Universidad de Sevilla, Edificio IBiS, E-14013 Seville, Spain

**Keywords:** Rho GTPases, RhoB, Cadherins, Adherens junctions, Prostate cancer

## Abstract

**Background:**

The Rho GTPase RhoB has been proposed to be a tumor suppressor in cancer and is downregulated in various tumors including prostate. RhoB has different effects on cell migration depending on the cell type and conditions, but the molecular basis for this variability is unclear. RhoB regulates trafficking of membrane receptors and integrins. We have previously shown that RhoB depletion alters focal adhesion dynamics and reduces surface levels of β1 integrin in PC3 prostate cancer cells, correlating with increased migration speed.

**Results:**

Here we show that RhoB depletion reduces cell-cell adhesion and downregulates E-cadherin levels as well as increasing internalized E-cadherin in DU145 prostate cancer cells. This is accompanied by increased migration speed. RhoB localizes to cell-cell junctions together with E-cadherin in DU145 cells. RhoB depletion also reduces N-cadherin levels in PC3 cells, which do not express E-cadherin.

**Conclusions:**

These results indicate that RhoB alters migration of cells with cell-cell adhesions by regulating cadherin levels. We propose that the relative contribution of integrins and cadherins to cell migration underlies the variable involvement for RhoB in this process and that the downregulation of RhoB in some epithelial cancers could contribute to the weakening of epithelial cell-cell junction during tumor progression.

**Electronic supplementary material:**

The online version of this article (doi:10.1186/s12964-015-0085-y) contains supplementary material, which is available to authorized users.

## Background

The Rho family of small GTPases are signalling molecules that regulate many cellular processes including cytoskeletal dynamics, cell motility, cell adhesion, cell division and transcription. They thereby contribute to wound healing, inflammation and cancer progression [1]. Most Rho family GTPases cycle between an active GTP-bound state and an inactive GDP-bound state. Their activation is controlled by guanine nucleotide exchange factors (GEFs) and GTPase activating proteins (GAPs), which activate or inactivate them respectively. In their active GTP-bound form, Rho GTPases interact with various downstream effectors to induce cellular responses.

RhoB, together with the closely related RhoA and RhoC, form the Rho subfamily within the Rho GTPase family. Despite the high sequence homology between these three proteins, RhoB has distinct biochemical and biological properties compared with RhoA and RhoC. RhoA and RhoC are modified at their C-terminus by the addition of a geranylgeranyl group, whereas RhoB can also be farnesylated. RhoB is the only Rho subfamily member that can be modified by palmitoylation [2,3]. RhoA and RhoC interact with RhoGDI, which extracts them from membranes by binding to the geranylgeranyl group and they are mostly localized in the cytoplasm. On the other hand, RhoB localizes mostly on the plasma membrane and/or on endosomes and does not bind to RhoGDI [4,5]. Consistent with its endosomal localization, RhoB regulates the trafficking of growth factor tyrosine kinase receptors through endosomes, including EGF receptor and VEGF receptor, and of the non-receptor tyrosine kinase Src, to the plasma membrane [6,7]. RhoB has also been described to localize to cell-cell junctions between Sertoli cells and germ cells in the testis [8].

RhoB has been postulated to act as a tumor suppressor in cancer and regulate apoptosis [9]. RhoB expression is reduced in several tumor types, including some prostate carcinomas, compared to non-cancer tissues and it is targeted by the miRNA miR21, involved in cancer progression [10,11]. RhoB expression is also induced by a variety of stresses including DNA damage, via JNK-mediated transcriptional upregulation [12,13]. RhoB overexpression inhibits proliferation, migration and invasion of gastric carcinoma cells [14]. On the other hand, mouse macrophages lacking RhoB, or human PC3 prostate cancer cells depleted of RhoB by RNAi, migrate faster than control cells. This correlates with reduced β integrin levels on the cell surface [15,16].

Epithelial cell-cell junction disruption occurs during progression of epithelial cancers [17]. E-cadherin is a homotypic cell-cell adhesion receptor that forms adherens junctions in epithelial cells and its localization to cell-cell contacts is dynamically regulated to control epithelial integrity during development and cancer progression [18].

Here we describe a new function for RhoB in maintaining cell-cell junctions in epithelial DU145 prostate cancer cells by regulating E-cadherin expression and localization. We also show that RhoB controls the levels of N-cadherin the mesenchymal-like PC3 prostate cancer cell line, which does not express E-cadherin. Decreased RhoB expression increases the migration of DU145 cells following the reduction in cell-cell adhesion.

## Results and discussion

### RhoB regulates cell-cell adhesion in epithelial cells

**Figure 1:**
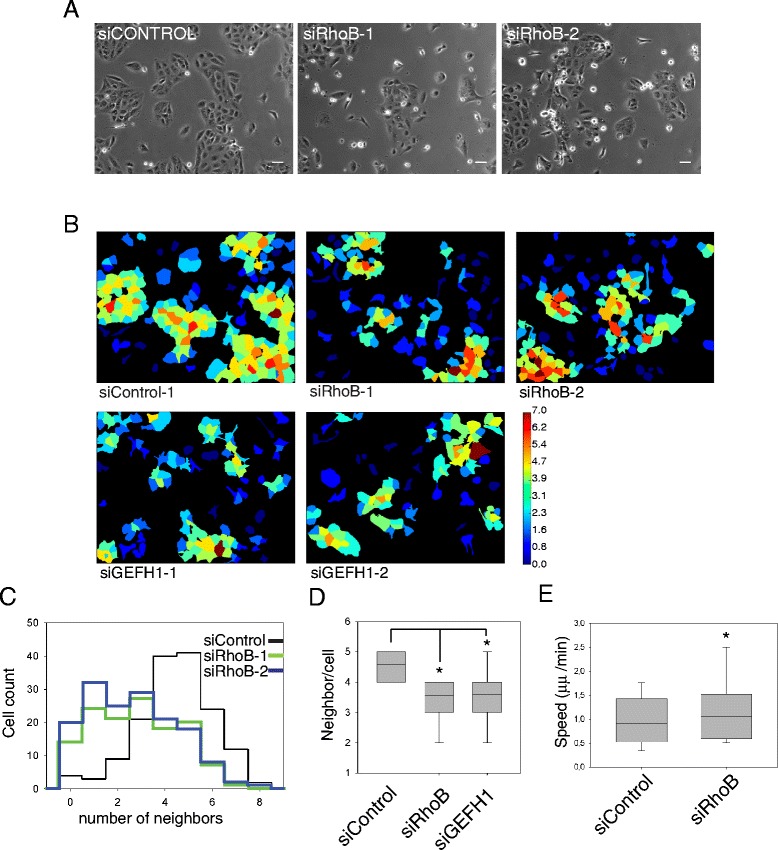
**RhoB knockdown affects colony formation in DU145 cells.** DU145 cells were transfected with the indicated siRNAs and analyzed after 72 h. **(A)** Phase contrast images of DU145 cells transfected as indicated. Bars 25 μm. (**B)** Representative images from Cell Profiler neighbor analysis. Cells are colored by the number of neighbors (i.e. adjacent cells), with higher numbers in warmer colors. **(C)** Histogram showing distribution of number of cell neighbors, determined from representative images shown in** A**. **(D)** Quantification of neighbors per cell determined from Cell Profiler neighbor analysis. Boxes of box and whisker plots show median, 25th and 75th percentile; whiskers show 95th percentile. Results are from 3 independent experiments with more than 150 cells per experiment and condition. Results from two different siRNA oligos for each gene were pooled. *p < 0.01. **(E)** Migration speed of DU145 cells on tissue culture-treated plastic. Cells were transfected with the indicated siRNAs. More than 50 cells per condition from 3 different experiments were tracked 58–60 h after transfection for 12–14 h. Boxes of box and whisker plots show median and 25th and 75th percentile; whiskers show 95th percentile. *p < 0.05.

We have previously shown that RhoB depletion by RNAi alters focal adhesions and reduces β1 integrin levels in PC3 prostate cancer cells. This correlates with increased migration speed of cells that predominantly move as single cells, such as macrophages and PC3 prostate cancer cells [15,16,19]. In addition to the plasma membrane and endosomes, RhoB has been reported to localize to cell-cell adhesions in some models [8]. To investigate whether RhoB could regulate cell-cell adhesions between epithelial cells, we depleted RhoB by RNAi in DU145 prostate cancer cells, which are epithelial in morphology and form colonies with E-cadherin-based adherens junctions [20,21]. siRNAs for RhoB were extensively used and characterized in our previous papers [15,22] and no change in RhoA or RhoC activity was observed after RhoB depletion. RhoB-depleted DU145 cells were less able to form colonies than control cells (Figure 1A and B). We quantified the effects of RhoB on cell-cell adhesion by performing a neighbor analysis that quantifies the average number of cells touching each individual cell in the image (neighbors). RhoB-depleted DU145 cells had a significant reduction in the number of cells forming cell-cell contacts with each other (Figure 1C and D). This change was accompanied by an increase in migration speed in the cell population (Figure 1E), mostly due to an increase in the migration of single cells not forming cell-cell contacts (Additional file 1). These changes were not due to a decrease in cell number as we have previously shown that RhoB knockdown does not affect cell proliferation [15]. Our previous analysis of PC3 cell morphology suggested that the RhoGEF GEF-H1 could act upstream to RhoB [15]. Interestingly, depletion of GEF-H1 also reduced cell-cell interaction (Figure 1B-D), suggesting that it plays a role in cell-cell interaction, potentially by regulating RhoB activity.

### RhoB alters E-cadherin expression and junctional maturation

**Figure 2:**
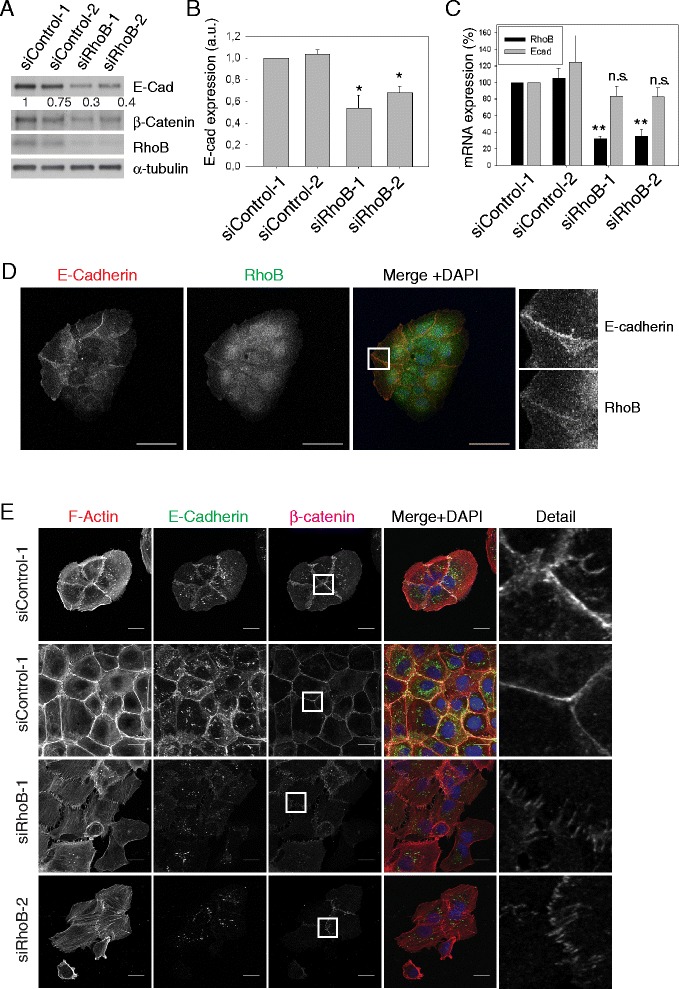
**RhoB regulates E-cadherin levels and cell-cell junction integrity. (A)** Western blot of lysates from DU145 cells transfected with the indicated siRNAs and probed with the indicated antibodies. Relative levels of E-cadherin expression normalized to α-tubulin loading control are shown below the blot. **(B)** Quantification of E-cadherin expression from western blots. Graph shows mean expression of E-cadherin +/− s.e.m. relative to siControl-1 from 4 different experiments; *p < 0.05. a.u. arbitrary units. **(C)** Results from RT-PCR showing mean relative mRNA expression (+/− s.d.; n = 3). **p < 0.005. n.s., not significant. **(D)** Immunofluorescence showing RhoB and E-cadherin staining in DU145 cells. Images on right show magnified images of the box marked in the merge panels. Bars 50 μm. **(E)** DU145 cells transfected with the indicated siRNAs were stained for F-actin and nuclei (DAPI in merge images) and with antibodies to E-cadherin and β-catenin. Detail panels show magnified images from the boxed regions in the β-catenin images. Bars 20 μm.

**Figure 3:**
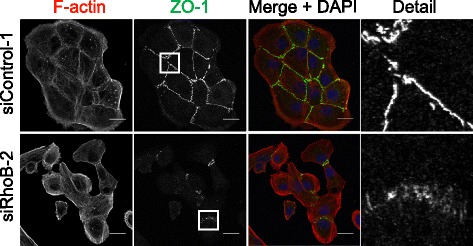
**RhoB affects cell-cell junctions.** DU145 cells transfected with the indicated siRNAs were stained for F-actin and nuclei (DAPI in merge) and with antibodies to ZO-1. Detail panels show magnified images from the boxed regions in the ZO-1 images. Bars 20 μm.

Because RhoB depletion reduced cell-cell interaction, we analyzed its effect on adherens junction components. RhoB depletion by RNAi reduced the expression of E-cadherin, a major component of adherens junctions, in DU145 cells (Figure 2A-C). No significant E-cadherin mRNA reduction was observed indicating that RhoB knockdown reduced E-cadherin expression at the protein level. We also observed a decrease in the levels of β-catenin, a cytoplasmic protein that interacts directly with E-cadherin [23], although we did not see an accumulation of β-catenin in the nucleus after RhoB knockdown. Interestingly endogenous RhoB could be partially localized to cell-cell adhesions in DU145 prostate epithelial cells, where it co-localized with E-cadherin (Figure 2D). This is consistent with our observation that RhoB regulates E-cadherin levels. The decrease in E-cadherin expression after RhoB depletion would be predicted to alter cell-cell junction organization. Together with the decrease in E-cadherin, we observed more disorganized ß-catenin localization at cell-cell contacts in RhoB-depleted cells that still conserved cell-cell adhesions (Figure 2E). β-catenin showed a ‘zipper’ distribution as described for early immature adherens junctions [24,25]. RhoB depletion also seemed to alter actin filament distribution: cells within colonies had reduced cortical actin filaments and instead had oriented stress fibers which appeared to connect to the discontinuous ‘zipper-like’ adherens junctions as described previously for endothelial cells [24]. At a later stage stress fibers are probably lost as observed most clearly in detached or more loosely adhered cells. These changes in stress fibers were not due to changes in RhoA activity after RhoB knockdown ([22] and data not shown) but could be a consequence of changes in cell-cell contact mediated tension. Similarly, ZO-1, a component of tight junctions [26], also showed ‘zipper’ localization in RhoB-depleted DU145 cells (Figure 3).

We propose that the effect of RhoB on cadherins could contribute to its tumor suppression function by participating in maintaining epithelial integrity. Reduced cadherin expression, due to a decrease in RhoB expression, increases migration in epithelial cancer cells.

### RhoB downregulation induces E-cadherin endosomal accumulation

**Figure 4:**
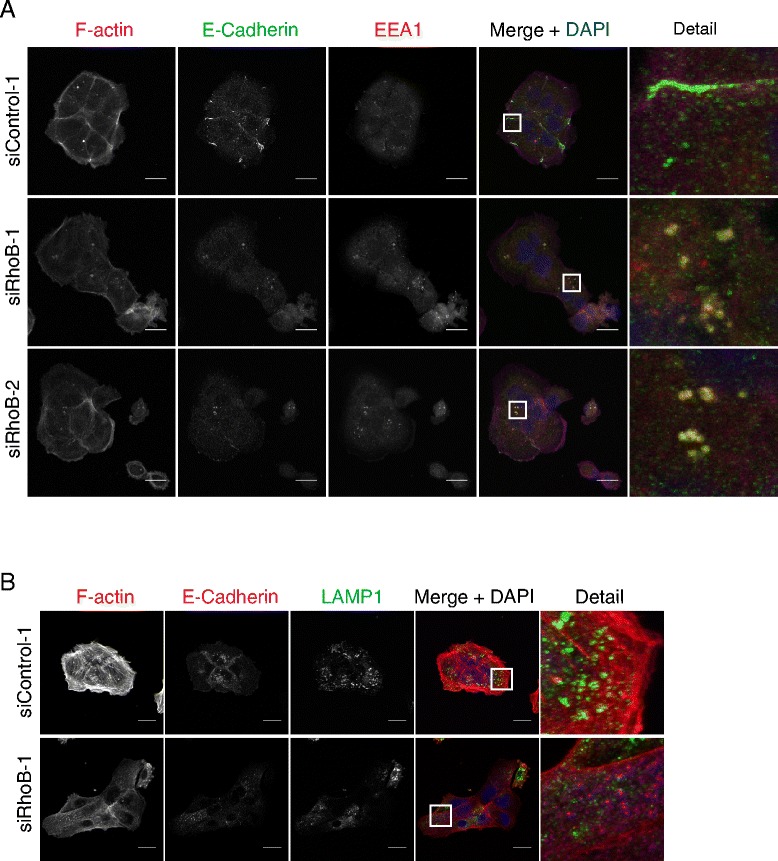
**E-cadherin accumulates in endosomes after RhoB knockdown. (A)** DU145 cells transfected with the indicated siControl or RhoB siRNAs were stained for F-actin and nuclei (DAPI in merge images) and with antibodies to E-cadherin and EEA1, an early endosome marker. Detail panel shows a magnified image from the boxed region in the merge image. Bars 20 μm. **(B)** DU145 cells transfected with the indicated siControl or RhoB siRNAs were stained for F-actin and nuclei (DAPI in merge) and with antibodies to E-cadherin and the lysosomal marker LAMP1. Detail panels show magnified images from the boxed regions in the merge images. Bars 20 μm.

E-cadherin endocytosis and recycling is important for cell-cell junction formation, turnover and maintenance [18]. RhoB can regulate endosomal trafficking and recycling of membrane receptors [6,7]. After RhoB depletion, the remaining E-cadherin accumulated in EEA1-positive vesicles, a marker for early endosomes (Figure 4A) indicating a problem in the recycling of E-cadherin from endosomes to the plasma membrane. Although RhoB can also localize to lysosomes [27], we did not observe co-localization of E-cadherin with the lysosomal marker LAMP1 (Figure 4B). These results indicate that RhoB contributes to cell-cell junction integrity in epithelial cells by regulating both E-cadherin expression and localization. It is likely that altered traffic of cadherins through endosomes by RhoB depletion ultimately increases cadherin degradation by targeting them to multivesicular bodies [28]. We did not observe significant cadherin localization in lysosomes, possibly because it had already largely been degraded by the time multivesicular bodies fused with lysosomes.

### RhoB regulates N-cadherin expression in PC3 cells

**Figure 5:**
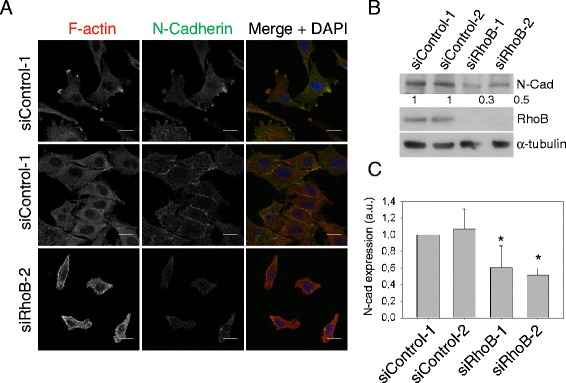
**RhoB reduces N-cadherin levels in PC3 cells. (A)** PC3 cells transfected with the indicated siRNAs were stained for F-actin and nuclei (DAPI in merge images), and with antibodies to N-cadherin. Bars 20 μm. **(B)** Western blot of cell lysates from PC3 cells transfected with the indicated siRNAs, and probed with the indicated antibodies. Relative levels of N-cadherin expression normalized to α-tubulin loading control are shown below the blot. **(C)** Quantification of N-cadherin expression. Graph shows mean expression of N-cadherin relative to siControl-1+/− s.e.m. from 4 different experiments; *p < 0.05. a.u., arbitrary units.

We have previously reported that RhoB regulates migration in PC3 prostate cancer cells by controlling focal adhesion dynamics and β1 integrin levels and localization [15]. N-cadherin expression levels can modulate cell migration and polarity by affecting integrin-based adhesions in tumor astrocytes [29]. As we have shown that RhoB regulates E-cadherin levels in epithelial cells, we investigated whether RhoB affects N-cadherin levels in cells where E-cadherin is not present. In control PC3 cells, which have undetectable levels of E-cadherin (data not shown), N-cadherin localized to actin-rich protrusions and to transient regions of cell-cell contact (Figure 5A), which are not very frequent in this mesenchymal cell line, as previously described [30]. RhoB depletion reduced N-cadherin levels in PC3 cells, and cells were rarely observed to interact with each other (Figure 5A-C). Consistent with this, timelapse movies indicated that RhoB-depleted PC3 cells formed fewer stable contacts with other cells when they touched (Additional file 2), which could reflect the reduced N-cadherin levels. However, N-cadherin depletion in PC3 cells did not induce the same morphological phenotype as RhoB depletion (data not shown), and thus it is unlikely that the effect of RhoB on cell shape [15] is due solely to altered N-cadherin levels. Integrin depletion and reduced adhesion to the substratum is probably the major mechanism underlying the observed phenotype [15] but N-cadherin downregulation could contribute to the increase in migration speed by reducing PC3 cell-cell adhesion.

Taken together, these results indicate that RhoB affects the expression and localization of cadherins and thereby alters cell-cell adhesion. RhoB has previously been described to regulate surface levels of EGF receptor and VEGF receptor and integrins [6,7,15,19]. Our work extends the types of membrane receptors regulated by RhoB to include cadherins. RhoB is often downregulated in prostate carcinomas compared with normal prostate tissues (Oncomine; www.oncomine.org). Although RhoB knockout mice do not have a clear defect in epithelial cell interactions [31], they have not been tested in models where increased junctional turnover would be revealed, for example in epithelial cancer progression models. RhoB knockout mice nevertheless are more susceptible to carcinogen-induced skin tumors [31] although the possible involvement of E-cadherin downregulation has not been tested in this model. We propose that the effect of RhoB on cadherins could contribute to its tumor suppressor function by participating in maintaining epithelial integrity. Reduced cadherin expression, due to a decrease in RhoB expression, increases migration in epithelial cancer cells. In epithelial-derived cancer cells that have lost or lack E-cadherin expression, decreased RhoB could also promote migration by its effect on integrins [15].

## Conclusions

The results presented here show that RhoB can affect levels and localization of cadherins in prostate cancer cells, thus participating in the maintenance of epithelial integrity. Reduced cadherin levels induced by RhoB downregulation lead to increased migration of cells that normally have an epithelial morphology with stable cell-cell junctions. In cells with epithelial origin which have lost E-cadherin expression but express N-cadherin, its regulation by RhoB could also contribute to a more migratory phenotype. Our work extends the types of membrane receptors regulated by RhoB to include cadherins and indicates a mechanism by which RhoB downregulation can contribute to tumor progression in prostate cancer.

## Methods

### Cell lines and reagents

DU145 and PC3 prostate cancer cell lines were grown in RPMI containing 25 mM Hepes and 2 mM glutamine supplemented with 10% FCS, 100 μg/ml streptomycin and 100 units/ml penicillin. The following antibodies were used: rabbit polyclonal (Santa Cruz 119, sc-180) or mouse monoclonal (Cell Signaling) RhoB antibodies, α-tubulin DM1A clone (Sigma, T6199), mouse monoclonal (BD Transduction, 610181) or rabbit polyclonal (Cell Signaling) E-cadherin, N-cadherin (BD Transduction), ZO-1 (Invitrogene), EEA1 (Santa Cruz), LAMP1 (Santa Cruz) and β-catenin (BD Transduction, 610153). Secondary HRP-labelled antibodies were from Amersham. Protease inhibitor cocktail (complete) was from Roche and Phosphatase inhibitor cocktail II and IV from Calbiochem.

### Transfections and western blotting

All siRNAs were from Dharmacon (GE Healthcare): siRhoB-1 (CAUCCAAGCCUACGACUAC), siRhoB-2 (GCAUCCAAGCCUACGACUA), on target plus siControl non-targeting siRNAs 1 and 2 (D-001810-01, D-001810-02), GEFH1-1 (CAACAUUGCUGGACAUUUC), GEFH1-2 (GAAUUAAGAUGGAGUUGCA). All siRNAs were initially tested for knockdown of the relevant protein in cells. Cell transfection, lysate preparation and immunoblotting were performed as described previously [22]. Cell lysates for western blot analysis were prepared 72 h after siRNA transfection.

### Timelapse microscopy

For phase contrast timelapse microscopy, a fully motorized, multi-field Nikon TE2000 microscope was used. Cell migration experiments were carried out as described [22]. Unless indicated, cells were imaged in medium containing 1% FCS.

### Confocal microscopy and cell shape and neighbor analysis

Immunofluorescence staining was carried out as previously described [22]. Alexa Fluor phalloidin (wavelengths 480 nm, 543 nm or 633 nm, Molecular Probes) was used for F-actin visualization, DAPI for nuclear staining, FITC-labelled α-tubulin antibody (DM1A clone) for microtubule staining and primary antibodies followed by Alexa Fluor-conjugated secondary antibodies (Molecular Probes) for ZO-1, β-catenin, E-cadherin, RhoB, LMP-1 or EEA1 stainings. Confocal images were acquired with a Zeiss LSM510 inverted confocal microscope. Morphology analysis was carried out using Metamorph or Cell Profiler software [32] from F-actin-stained fluorescence images. For neighbor analysis DU145 cells were transfected with siRNA and seeded at subconfluence. Cultures were left to grow for 72 h before fixing and staining for F-actin and DAPI. Neighbor analysis was performed with Cell Profiler [32] from fluorescence images.

### RT-PCR

Total RNA was extracted from DU145 cells 50 h post siRNA transfection (RNeasy MICRO kit; Qiagen) and reverse transcribed with the QuantiTec Reverse transcription kit (Qiagen) according to the manufacturer’s instructions. Quantitative real time PCR was performed with 20 ng of cDNA in a ViiA7 thermocycler using SYBR Green detection system (Applied biosystems). Data analysis was performed with the ΔCt method. Specific oligonucleotides were used for the detection of E-cadherin (TCGACACCCGATTCAAAGTG, GTCCCAGGCGTAGACCAAGA) or RhoB (CCCACCGTCTTCGAGAACTA, CACCGAGAAGCACATGAGAA) and GAPDH oligonucleotides (GTGAAGGTCGGAGTCAACG, TGAGGTCAATGAAGGGGTC) were used for loading control.

### Statistical analysis

For statistical significance analysis either an unpaired two-tailed t-test (for comparing two conditions) or a One-way ANOVA with Dunn’s Multiple Comparison post Test (for comparing multiple conditions) was used. All significances indicated are compared to siControl condition unless stated. At least 2 different siRNAs for each gene were analysed in every assay and results from the 2 siRNAs were pooled in some graphs.

## Additional files

Additional file 1:
**DU145 cell migration.** Timelapse movie of DU145 cells transfected with siControl-1 siRNA or siRhoB-2 siRNA. siRhoB transfected cultures have more single cells that migrate faster. Bar 50 μm. Additional file 2:
**PC3 cell-cell contacts.** Timelapse movie of PC3 cells transfected with siControl-1 siRNA or siRhoB-2 siRNA. siRhoB transfected cells are less prone to form stable cell-cell contacts when they touch than control cells. Bar 50 μm.

## Abbreviations

GEF: Guanine nucleotide exchange factor
GAP: GTPase activating protein
RhoGDI: Rho GDP-dissociation inhibitor
JNK: Jun N-terminal kinase
FCS: Fetal calf serum
RT-PCR: Real time polymerase chain reaction
